# Only the Rye Derived Part of the 1BL/1RS Hybrid Centromere Incorporates CENH3 of Wheat

**DOI:** 10.3389/fpls.2021.802222

**Published:** 2021-12-13

**Authors:** Raheleh Karimi-Ashtiyani, Veit Schubert, Andreas Houben

**Affiliations:** ^1^Department of Biotechnology, Faculty of Agriculture, Tarbiat Modares University, Tehran, Iran; ^2^Leibniz Institute of Plant Genetics and Crop Plant Research (IPK), Gatersleben, Germany

**Keywords:** CENH3, 1BL/1RS, Robertsonian translocation, wheat, dicentric, rye, hybrid centromere

## Abstract

The precise assembly of the kinetochore complex at the centromere is epigenetically determined by substituting histone H3 with the centromere-specific histone H3 variant CENH3 in centromeric nucleosomes. A chromosome 1B reconstructed in wheat by centric misdivision from two wheat-rye centric translocations is known to carry a hybrid wheat-rye centromere. The resulting hybrid (dicentric)centromere is composed of both wheat and rye centromeric repeats. As CENH3 is a marker for centromere activity, we applied Immuno-FISH followed by ultrastructural super-resolution microscopy to address whether both or only parts of the hybrid centromere are active. Our study demonstrates that only the rye-derived centromere part incorporates CENH3 of wheat in the 1BL/1RS hybrid centromere. This finding supports the notion that one centromere part of a translocated chromosome undergoes inactivation, creating functional monocentric chromosomes to maintain chromosome stability.

## Introduction

The centromere is the assembly site for the proteinaceous kinetochore complex, which enables the proper segregation of sister chromatids and their transmission to the daughter cells during mitosis and meiosis. Despite the conserved function of the centromere, the primary constriction of monocentric species is generally enriched in highly diverged gene-free repetitive sequences. These repeats include satellite DNAs and retrotransposons (Barra and Fachinetti, 2018; Talbert and Henikoff, 2020). The centromeric DNA sequence is not sufficient to maintain the centromere position and function. The precise assembly of the kinetochore complex at the centromere is epigenetically determined by substituting a centromere-specific histone H3 variant CENH3 for histone H3 in centromeric nucleosomes (Henikoff and Dalal, 2005; Allshire and Karpen, 2008). Besides some diploid species encoding multiple functional CENH3 variants, e.g., barley (Sanei et al., 2011; Ishii et al., 2015), rye (Evtushenko et al., 2017, 2021), *Fabeae* species (Neumann et al., 2015), and cowpea (Ishii et al., 2020), most diploid eukaryotes encode only one variant of CENH3. In contrast, in allopolyploids species like wheat (Yuan et al., 2015), each parental subgenome possesses at least one CENH3 variant operating in the context of multiple species-specific centromeric sequences.

Defects in the pathways that regulate centromere assembly and function affect the proper chromosome segregation and lead to chromosome instability and lethality in various organisms (Maheshwari et al., 2015; Britt and Kuppu, 2016; Barra and Fachinetti, 2018). Also, the alteration of CENH3 that impairs its loading and maintenance results in chromosome missegregation (Au et al., 2008; Pidoux et al., 2009; Ravi and Chan, 2010; Karimi-Ashtiyani et al., 2015; Kuppu et al., 2015, 2020; Shrestha et al., 2017; Karimi-Ashtiyani, 2021).

Due to the high density of repetitive sequences and high similarity to non-homologous centromeres within a species, the primary constriction is vulnerable to breakage and recombination (Sullivan et al., 1996; Friebe et al., 2005; Barra and Fachinetti, 2018). Therefore, chromosome rearrangements and breaks often involve (peri)centromeric regions (Friebe et al., 2005; Lukaszewski, 2010; Barra and Fachinetti, 2018). Centric misdivisions, followed by the fusion of the broken arms from different chromosomes, result in whole-arm Robertsonian translocations (Robertson, 1961), which usually possess centromeric sequences of both chromosomes (Zhang et al., 2001; Song et al., 2016). However, to ensure mitotic stability, these chromosomes having a “hybrid (dicentric)centromere” convert to functionally monocentric chromosomes where only one centromere part remains active (Friebe et al., 2005; Han et al., 2006). It is suggested that centromere inactivation involves genomic and/or epigenomic reprogramming (Stimpson et al., 2012). Chromosome breaks and fusions occurred at centromeric and pericentromeric regions in wheat-rye translocation lines (Lukaszewski, 1997; Zhang et al., 2001; Friebe et al., 2005). The most common centric translocation is the 1BL/1RS translocation, in which the short arm of wheat chromosome 1B is replaced by the short arm of rye chromosome 1R (Lukaszewski, 2010; Moskal et al., 2021). Various 1BL/1RS translocations have served as valuable stocks in wheat breeding programs as chromosome 1R contains insect and disease resistance genes (Kim et al., 2004)^1^.

To address whether both or only one part of the 1BL/1RS hybrid centromere is active Wang et al. (2017) applied a combination of immuno-FISH, chromatin immunoprecipitation (ChIP)-qPCR, and RT-PCR. They concluded that all analyzed 1BL/1RS translocations contain hybrid centromeres and that the wheat-derived CENH3 is incorporated into both, the wheat- and rye-derived components of the hybrid centromere. The application of wheat and rye centromere-specific repeats, like the *CRW* clone 6C6 (Wang et al., 2017) and *pAWRC.1/Bilby* (Francki, 2001), respectively, allowed the visualization of the 1BL/1RS hybrid centromere. Both repeats are part of the CENH3-positive chromatin in either wheat (Liu et al., 2008; Li et al., 2013) or rye (Houben et al., 2011). None of the rye CENH3 variants is encoded by the short arm chromosome 1R (Evtushenko et al., 2021; Rabanus-Wallace et al., 2021). Hence, the 1BL/1RS-located CENH3 derives from wheat.

We extended the analysis and applied ultrastructural super-resolution microscopy to analyze the arrangement of the 1BL/1RS centromere at a resolution of ∼120 nm. Surprisingly, our findings demonstrate that only the rye-derived centromere incorporates CENH3 of wheat into the hybrid centromere of 1BL/1RS.

## Materials and Methods

### Plant Material and Cultivation

The 1B_rec_-1 line of cv. Pavon 76 carrying a reconstructed chromosome 1B (Lukaszewski, 1993, 1997) was grown in a greenhouse at 16 h light, 22°C day/16°C night conditions. Chromosome 1B_rec_-1 was reconstructed by centric misdivision from two centric wheat-rye translocations, 1RS.1BL and 1BS.1RL. In essence, the chromosome itself is a centric translocation, composed of a wheat chromosome arm 1BS from cv. Pavon 76 and 1BL arm from the translocation 1RS.1BL from the Aurora/Kavkaz source (Lukaszewski, 1993, 1997). As demonstrated by Zhang et al. (2001) this chromosome carries a hybrid centromere, composed in part of a wheat centromere and in part of a rye centromere.

### Fluorescence *in situ* Hybridization and Indirect Immunostaining

Root tips and anthers of five individual plants of line 1BS_PVN_.1BL_VEE_ were used for the preparation of mitotic and meiotic chromosomes, according to Gernand et al. (2003). Immunostaining followed by FISH was done as described by Ma et al. (2010). The rye centromere-specific repeat *Bilby* (Francki, 2001), and the wheat centromere-specific repeat *CRW2* (Liu et al., 2008) labeled by nick translation with Alexa 488-dUTP (Invitrogen), and Cy5-dUTP (GE Healthcare Life Sciences), respectively, were used as FISH probes. The primary antibodies, rabbit anti-Os-CENH3 (Nagaki et al., 2004), and the secondary antibodies, rhodamine-conjugated anti-rabbit IgG (Dianova) were used for indirect immunostaining. Finally, the slides were mounted in antifade (Vectashield) containing 4,6-diamidino2-phenylindole (DAPI) as counterstain.

### Microscopy

Imaging was performed by using an Olympus BX61 microscope and an ORCA-ER CCD camera (Hamamatsu). Deconvolution microscopy was employed for superior optical resolution of spherical structures. To investigate the centromeric chromatin ultrastructures at an optical resolution of ∼120 nm (super-resolution achieved with a 488 nm laser excitation), we applied spatial structural illumination microscopy (3D-SIM) using a 63/1.40 objective of an Elyra PS.1 super-resolution microscope system (Carl Zeiss GmbH), (Weisshart et al., 2016). 3D-SIM image stacks were used to perform surface rendering and to produce the movies via the Imaris 9.7 (Bitplane) software.

## Results and Discussion

**FIGURE 1:**
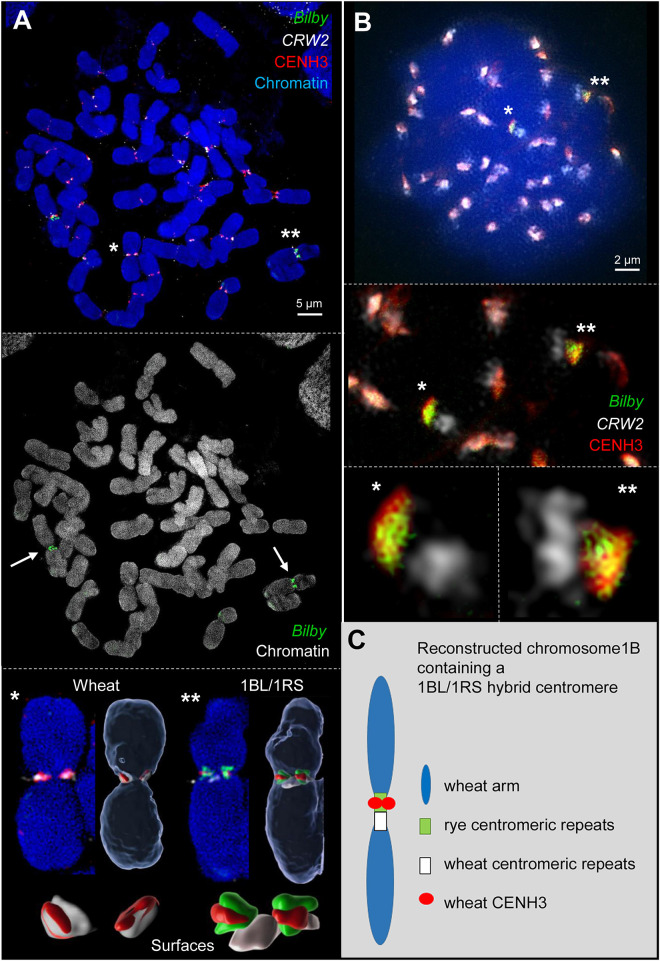
Wheat CENH3 incorporates exclusively into rye centromere repeats containing chromatin of the 1BL/1RS hybrid centromere during mitosis **(A)** and meiosis **(B)** as revealed by 3D-SIM. **(A)** Somatic wheat metaphase cell containing two 1BL/1RS homologs labeled by rye-specific *Bilby* repeats (arrows). All wheat chromosomes incorporate wheat CENH3 into the wheat-specific centromeric *CRW2* repeat-positive chromatin (*). Instead, wheat CENH3 loads only into the rye-specific *Bilby* repeat-positive chromatin of the translocated 1BL/1RS chromosomes (**). Surface rendering of the centromeric components (below) show clearly the different co-localizations (see also Supplementary Movies 1, 2). **(B)** Early meiotic metaphase I showing in addition to all labeled wheat centromeres both 1BL/1RS homologs labeled specifically by *Bilby* (asterisks). During meiosis wheat CENH3 places also exclusively into the rye derived centromeric chromatin as shown enlarged below. The co-localization of CENH3 and *Bilby* induces the yellow color. **(C)** Schematic representation of the reconstructed wheat chromosome 1B with a hybrid wheat-rye centromere. This centromere is composed of wheat and rye centromeric repeats. The wheat-derived CENH3 co-localized only with the rye-derived centromeric chromatin creating a functional chromosome.

Fluorescence *in situ* hybridization with the rye- and wheat-specific centromere repeats *pAWRC.1/Bilby* (Francki, 2001) and *CRW2* (Liu et al., 2008), respectively, demonstrated the hybrid structure of the 1BL/1RS centromere of the translocation line 1BS_PVN_.1BL_VEE_ at somatic metaphase chromosomes (Figure 1A and Supplementary Figure 1). In line with previous publications (Lukaszewski, 1997; Zhang et al., 2001) one half of the hybrid centromere displayed rye and the other half wheat centromere-specific signals.

As CENH3 is a mark for centromere activity (Talbert and Henikoff, 2020), the hybrid 1BL/1RS centromere activity was determined by immunostaining using antibodies that cross-reacts with the CENH3 proteins of cereals (Nagaki et al., 2004; Liu et al., 2008; Houben et al., 2011). Subsequent FISH with differentially labeled centromeric repeats *Bilby* (Francki, 2001) and *CRW2* (Liu et al., 2008) was performed to address whether the entire hybrid centromere or only the wheat- or rye- derived centromere component is active. In all analyzed dividing cells (about 80) of five plants, CENH3 signals consistently co-localized with the wheat centromeric repeat *CRW2* in all non-translocation chromosomes. In contrast, only the rye-derived centromere of 1BL/1RS co-localized with the CENH3 signals (Figure 1A and Supplementary Figure 1). To confirm this observation, naturally extended meiotic prophase I chromosomes were labeled and analyzed by super-resolution microscopy (Figure 1B). Again, the rye derived centromere component co-localized with CENH3 signals. The *CRW2-*positive wheat centromeric region of 1BL/1RS was free of CENH3. The results are summarized in Figure 1C.

Our observation differs from the findings described by Wang et al. (2017) who described that CENH3 binds to both components of the 1BL/1RS hybrid centromere. What might be the reason for this difference? The apparent difference between both studies might be caused by the different 1BL/1RS genotypes analyzed and the different microscopical resolution achieved (diffraction-limited versus super-resolution microscopy). In addition, the ChIP-qPCR experiment performed by Wang et al. (2017) confirmed the interaction of the rye centromeric repeat *pAWRC*.1/*Bilby* with CENH3-containing nucleosomes. Still, it could not address whether the wheat component of the 1BL/1RS hybrid centromere interacts with CENH3 too. Besides, in their colocalization study of centromeric repeats with CENH3, they solely used a rye centromeric-specific probe without including any wheat centromere-specific probe. As only a fraction of CENH3 signals co-localized with the rye-specific probe, the authors concluded that CENH3 interacts with wheat centromeric sequences as well. In our study, we differentially labeled both centromeric repeats of rye and wheat (*Bilby* and *CRW2*) together with CENH3 in a single immuno-FISH experiment.

Although the centromere position is epigenetically determined and does not depend on its underlying sequences, studies in maize (Zhong et al., 2002), rice (Nagaki et al., 2005), wheat-rye B addition lines (Banaei-Moghaddam et al., 2012) and many other species have shown that CENH3 containing nucleosomes bind differentially to the repeats of the centromeres. Why the rye- and not the wheat-derived centromere component of the 1BL/1RS chromosome is epigenetically marked by CENH3 is unknown. However, it is tempting to speculate that fusion of the chromosome arm 1BL with 1RS triggered centromere sequence changes and, therefore CENH3 preference. The centromeric sequences of hybrids between distantly related or translocated chromosomes may undergo rearrangements like expansion and reduction. For example, centromeric retrotransposons were strongly reduced or eliminated in wheat aneuploids and addition lines from wide hybrids (Guo et al., 2016). The presence of CENH3 signals at novel positions suggests a *de novo* centromere formation. In contrast, rye-specific centromeric sequences in wheat-rye hybrids expanded towards the chromosome arms, possibly causing centromere expansion (Guo et al., 2016).

It is also possible that differential methylation of centromeric repeats may underlay the inactivation of wheat centromeric repeats in the hybrid centromere of 1BL/1RS. Investigations of the epigenetic landscape for the centromeres in Arabidopsis and maize showed that the centromere repeats associated with CENH3 are hypomethylated compared to flanking centromere repeats (Zhang et al., 2008).

In summary, our analysis of a 1BL/1RS centromere provides an additional example that one component of a hybrid (dicentric)centromere undergoes inactivation creating functional monocentric chromosomes to maintain their stability. Further investigations are needed to determine whether the fusion of 1BL with 1RS triggered centromere sequence changes and or epigenetic modifications and consequently CENH3 preference.

## Data Availability Statement

The original contributions presented in the study are included in the article/Supplementary Material, further inquiries can be directed to the corresponding authors.

## Author Contributions

AH designed the study and provided editorial input to the manuscript. RK-A and VS performed the research and interpreted the results. RK-A wrote the first draft of the manuscript. All authors contributed to manuscript revision and approved the submitted version.

## Conflict of Interest

The authors declare that the research was conducted in the absence of any commercial or financial relationships that could be construed as a potential conflict of interest.

## Publisher’s Note

All claims expressed in this article are solely those of the authors and do not necessarily represent those of their affiliated organizations, or those of the publisher, the editors and the reviewers. Any product that may be evaluated in this article, or claim that may be made by its manufacturer, is not guaranteed or endorsed by the publisher.

## Footnotes

1
https://www.rye-gene-map.de/rye-introgression/html/preface.html

## Supplementary Material

Supplementary Figure 1 Wheat CENH3 incorporates exclusively into rye centromere repeats containing chromatin of the 1BL/1RS hybrid centromere during mitosis **(A–F)** and meiosis **(G–L)** revealed by standard fluorescence microscopy. **(A–F)** Wheat metaphase cells containing 1BL/1RS translocations (arrows) after CENH3-immunostaining and FISH with rye (*Bilby*, in green) and wheat (*CRW2*, in white) centromere-specific repeats. CENH3 signals co-localized with *Bilby* (centromeric repeat of rye) and not with the centromeric repeats of wheat in 1BL/1RS. **(G–L)** Meiotic metaphase I showing in addition to all labeled wheat centromeres a 1BL/1RS bivalent (arrow). **(C,I)** Co-localization of CENH3 and *CRW2*. **(D,J)** Co-localization of CENH3 and *Bilby*. **(E,K)** FISH with rye and wheat centromere-specific repeats (*Bilby* and *CRW2*, respectively). **(F,L)** Further enlarged views of 1RS/1BL. Selected chromosomes are labeled by *.

Supplementary Movie 1 Wheat CENH3 (red) co-localizes the wheat-specific CRW2 repeats (white) of a wheat centromere.

Supplementary Movie 2 Wheat CENH3 (red) co-localizes with the rye-specific centromeric Bilby repeats (green), but not with the wheat-specific CRW2 repeats (white) of a hybrid centromere of a translocated 1BL/1RS chromosome.
