# Corrigendum: Only the Rye Derived Part of the 1BL/1RS Hybrid Centromere Incorporates CENH3 of Wheat

**DOI:** 10.3389/fpls.2022.854911

**Published:** 2022-03-04

**Authors:** Raheleh Karimi-Ashtiyani, Veit Schubert, Andreas Houben

**Affiliations:** ^1^Department of Biotechnology, Faculty of Agriculture, Tarbiat Modares University, Tehran, Iran; ^2^Leibniz Institute of Plant Genetics and Crop Plant Research (IPK), Gatersleben, Germany

**Keywords:** CENH3, 1BL/1RS, Robertsonian translocation, wheat, dicentric, rye, hybrid centromere

In the original article, there was an error. The origin and history of the analysed 1B/1R hybrid centromere was not correctly described and required correction in the **Abstract, Materials and Methods section, and Figure 1C**.

A correction has been made to the **Abstract**.

The original text, “The wheat-rye 1BL/1RS translocation chromosome in the background of wheat resulted from a centric misdivision followed by the fusion of the broken arms of chromosomes 1B and 1R from wheat and rye, respectively” has been corrected to: “A chromosome 1B reconstructed in wheat by centric misdivision from two wheat-rye centric translocations is known to carry a hybrid wheat-rye centromere.”

A correction has been made to the section **Materials and Methods**, subsection **Plant Material and Cultivation**, paragraph 1:

“The 1B_rec_-1 line of cv. Pavon 76 carrying a reconstructed chromosome 1B (Lukaszewski, 1993, 1997) was grown in a greenhouse at 16 h light, 22°C day/16°C night conditions. Chromosome 1B_rec_-1 was reconstructed by centric misdivision from two centric wheat-rye translocations, 1RS.1BL and 1BS.1RL. In essence, the chromosome itself is a centric translocation, composed of a wheat chromosome arm 1BS from cv. Pavon 76 and 1BL arm from the translocation 1RS.1BL from the Aurora/Kavkaz source (Lukaszewski, 1993, 1997). As demonstrated by Zhang et al. (2001) this chromosome carries a hybrid centromere, composed in part of a wheat centromere and in part of a rye centromere.”

In the original article, there was a mistake in Figure 1C as published because the origin of the chromosome arms was wrongly depicted. Additionally, the origin of the analysed chromosome was wrongly described in the legend for Figure 1C. The corrected Figure 1C and its legend appears below.

**Figure 1:**
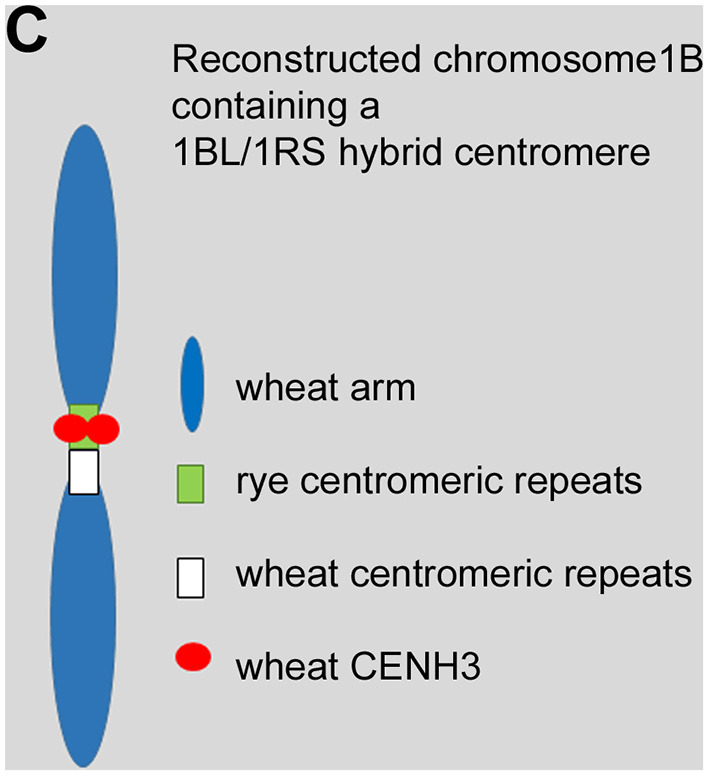
**(C)** Schematic representation of the reconstructed wheat chromosome 1B with a hybrid wheat-rye centromere. This centromere is composed of wheat and rye centromeric repeats. The wheat-derived CENH3 co-localized only with the rye-derived centromeric chromatin creating a functional chromosome.

The authors apologize for these errors and state that this does not change the scientific conclusions of the article in any way. The original article has been updated.

## Publisher's Note

All claims expressed in this article are solely those of the authors and do not necessarily represent those of their affiliated organizations, or those of the publisher, the editors and the reviewers. Any product that may be evaluated in this article, or claim that may be made by its manufacturer, is not guaranteed or endorsed by the publisher.
