# Supplementary figures and images for: Only the Rye Derived Part of the 1BL/1RS Hybrid Centromere Incorporates CENH3 of Wheat
Source: Front Plant Sci. 2021 Dec 13;12:802222. doi: 10.3389/fpls.2021.802222 (PMC8710534; doi:10.3389/fpls.2021.802222)

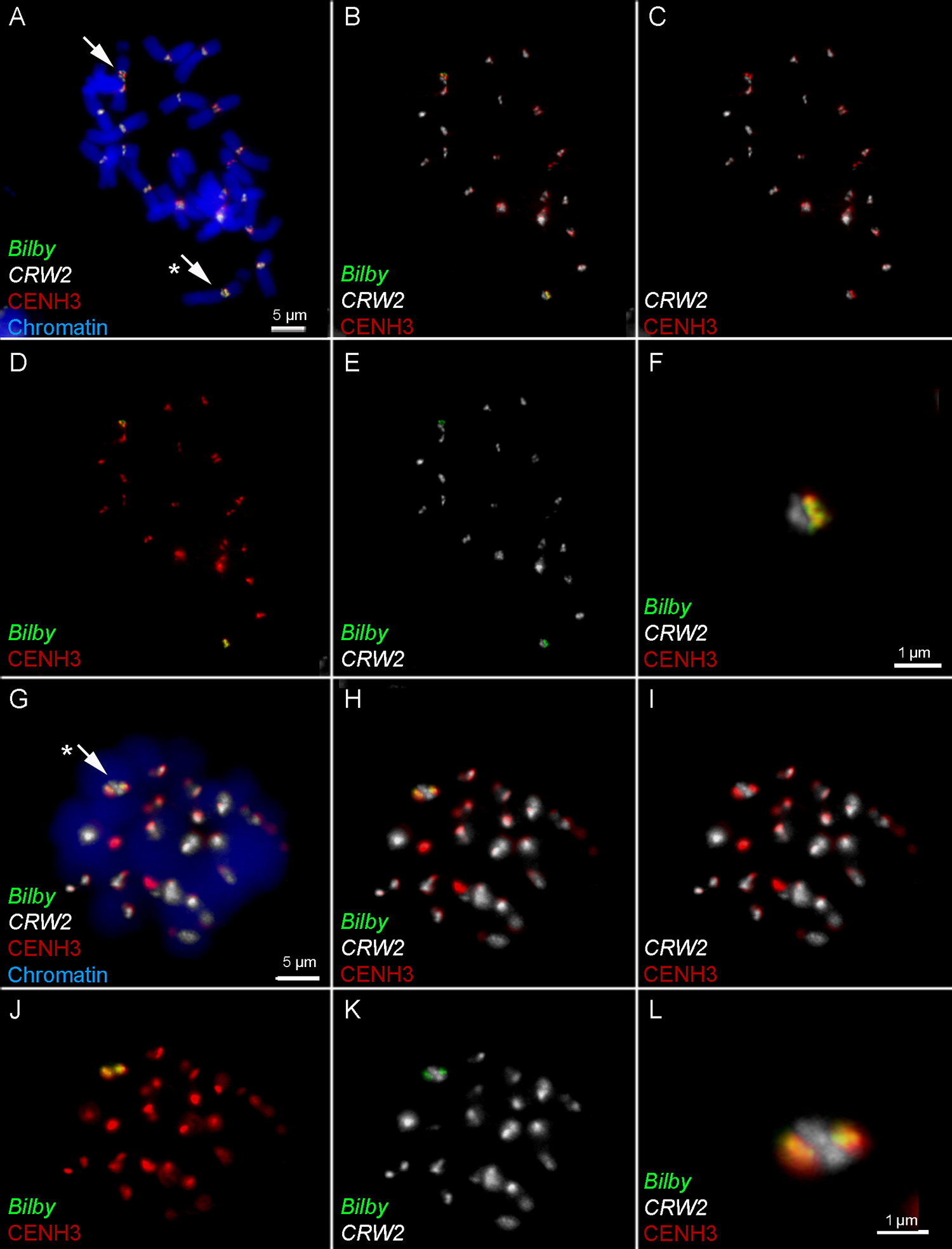

Supplement: Supplementary Figure 1 — Wheat CENH3 incorporates exclusively into rye centromere repeats containing chromatin of the 1BL/1RS hybrid centromere during mitosis (A–F) and meiosis (G–L) revealed by standard fluorescence microscopy. (A–F) Wheat metaphase cells containing 1BL/1RS translocations (arrows) after CENH3-immunostaining and FISH with rye (Bilby, in green) and wheat (CRW2, in white) centromere-specific repeats. CENH3 signals co-localized with Bilby (centromeric repeat of rye) and not with the centromeric repeats of wheat in 1BL/1RS. (G–L) Meiotic metaphase I showing in addition to all labeled wheat centromeres a 1BL/1RS bivalent (arrow). (C,I) Co-localization of CENH3 and CRW2. (D,J) Co-localization of CENH3 and Bilby. (E,K) FISH with rye and wheat centromere-specific repeats (Bilby and CRW2, respectively). (F,L) Further enlarged views of 1RS/1BL. Selected chromosomes are labeled by *. [file Image_1.TIF]
